# The Blood: How to Examine and Diagnose Its Diseases

**Published:** 1899-03

**Authors:** 


**The Blood: How to Examine and Diagnose its Diseases.** By ALFRED C. COLES, M.D. Pp. xii., 260. London: J. & A. Churchill. 1898.

The scope of this book is limited to the examination and staining of the formed elements of the blood. The practical directions for the preparation and staining of blood-films are full and clear enough to enable anyone who carefully follows them to procure good specimens. Dr. Coles advocates the use of hæmatoxylin and eosin as stains, and the plates show that in his hands this method has given good results. We agree that the method of staining with eosin and methylene blue is difficult to carry out, and somewhat uncertain in its results. The author also advises alcohol and ether instead of heat for fixing blood-films. Sections on the pathology of the diseases described are given, but are too short to allow of an adequate treatment of the subject. Generally speaking, the references to the literature are full, and the opinions of other writers given at length. We think that the author might have given his own views more decidedly, and in some cases he has left his statements too vague for a text-book, but this well-printed volume, taken as a whole, gives a satisfactory account of the subject, and will no doubt be found useful for aid in work and for reference. The plates are well executed in colours. An illustration of the Thoma-Zeiss hæmacytometer would render the description more complete.

